# Spatial population study on the association among socio-economic indicators and oral health in preschool children in Buenos Aires

**DOI:** 10.1136/bmjopen-2026-116230

**Published:** 2026-05-20

**Authors:** Aldo Squassi, Eliana Belén González, Fiorella Ventura, Rocio Lazzati, Glenda Rossi, Pablo Salgado, Andrea Cappai, Marco Dettori, Guglielmo Campus

**Affiliations:** 1 Instituto de Investigaciones en Salud Pública, Cátedra de Odontología Preventiva y Comunitaria, Universidad de Buenos Aires Facultad de Odontología, Buenos Aires, Argentina; 2 Consejo Nacional de Investigaciones Científicas y Tecnológicas (CONICET), Buenos Aires, Argentina; 3 Department of Architecture, Design and Urban Planning, University of Sassari, Alghero, Italy; 4 Department of Medicine, Surgery and Pharmacy, University of Sassari, Sassari, Italy; 5 Deptartment of Cariology, University of Gothenburg Sahlgrenska Academy, Gothenburg, Sweden; 6 Department of Dental Science and Maxillo-Facial Surgery, Università degli Studi di Roma La Sapienza, Rome, Italy; 7 Department of Cariology, Saveetha, Dental College and Hospitals, SIMATS, Chennai, India

**Keywords:** Child, Dentistry, PUBLIC HEALTH, EPIDEMIOLOGY

## Abstract

**Abstract:**

**Objectives:**

Dental caries is the most prevalent chronic condition among Argentine children, with distribution and severity strongly shaped by social and territorial inequalities. This study evaluated caries treatment needs and their spatial and socio-economic associations among preschool children in Buenos Aires.

**Design, setting, participants:**

An ecological population study was conducted among 54 337 6-year-old children attending public schools in Buenos Aires. Caries severity was measured using the Caries Treatment Needs Index (CTNI) by calibrated examiners.

**Primary and secondary outcome measures:**

Socio-economic indicators included individual health coverage, neighbourhood housing prices, distance to the nearest primary health centre, population density and the proportion of households with unsatisfied basic needs. Analyses comprised descriptive statistics, multivariate regression, ORs and spatial autoregressive models.

**Results:**

Overall, 67.9% of children had treatment needs (CTNI >2) and 17.5% had high needs (CTNI >10), with significant heterogeneity across municipalities (p<0.01). Public health coverage showed a strong gradient with increasing caries severity (OR: 1.77–3.68; p<0.01). Lower housing prices, greater distance to health centres, higher population density and higher unmet basic needs were independently associated with worse CTNI outcomes (p<0.01). Spatial analyses confirmed significant territorial clustering of treatment needs.

**Conclusions:**

Caries treatment needs in Buenos Aires follow a clear socio-economic and spatial gradient, with both individual and neighbourhood disadvantage independently associated with increased needs and highlighting the need for targeted, territorially focused public health strategies.

**STRENGTHS AND LIMITATIONS OF THIS STUDY**

- Population-based survey across 432 public schools with a very large sample of 6-year-old children.
- Standardised Caries Treatment Needs Index examinations performed by 25 calibrated examiners.
- Use of multilevel socio-economic proxies at individual, family, school and neighbourhood levels.
- School geolocation used as a proxy for residence because individual home addresses were unobtainable.
- Ecological spatial modelling may be affected by ecological fallacy and the modifiable areal unit problem.

## Introduction

 The notion that disease aetiology and distribution can be attributed to causes categorised as distant or proximate (including internal) has a long historical precedent. In contrast, the concept of a causal etiological hierarchy, extending from distal to proximal, is a comparatively recent development, becoming a core component of public health curricula only in the mid-20th century.^1^ This principle is inherently applicable to predominant oral diseases, which are classified by the WHO as non-communicable diseases, although their incorporation into public health frameworks and related implications remain insufficiently discussed.^2^

Argentina is the third-largest economy in Latin America, with a gross domestic product of approximately US$620 billion.^3^,^5^ The country faces persistent macroeconomic challenges, including high inflation, currency devaluation, fiscal volatility and pronounced social inequalities. Its health system is a fragmented mix of public, social security (*Obras Sociales*) and private sectors. While the public system provides universal free access, it is often strained by long waiting lists; *Obras Sociales* are mandatory for formal workers, and the private sector serves those who can afford it.^6^

The socio-economic environment of Buenos Aires (Argentina’s capital) strongly impacts developmental and health outcomes, particularly among preschool children. In affluent neighbourhoods such as Palermo and Belgrano, children benefit from enriched early childhood education (ECE), stable housing and comprehensive healthcare, supporting optimal cognitive, emotional and physical development. Conversely, children in disadvantaged areas such as Villa Lugano, Soldati and southern peripheries face constrained access to quality preschool education, food insecurity and overcrowding. These conditions are associated with higher rates of respiratory infections, malnutrition and developmental delays.^7^ Health disparities are exacerbated by unequal access to healthcare, as public health centres in deprived areas are often under-resourced, leading to delayed diagnosis and treatment. A 2023 UNICEF Argentina report noted that children in these areas are less likely to receive routine vaccinations and preventive care, increasing avoidable illness.^8^ Addressing these disparities requires integrated policies to expand access to quality ECE, strengthen primary healthcare in vulnerable areas and support families through income and nutrition programmes.

The consequences of poor oral health in children extend beyond pain and infection, necessitating multipronged strategies such as school-based dental programmes, increased public investment and integration of oral health education into primary care and early childhood services.^9^,^12^ In Argentina, dental caries remains the most prevalent chronic disease in children, affecting over 50% of those aged 5–11, with higher prevalence in low-income and rural populations (Pan American Health Organization, 2024).^13 14^ These disparities are closely linked to socio-economic status, diet and access to preventive dental care, while access to oral healthcare remains uneven nationwide.

The Caries Treatment Needs Index (CTNI) is a standardised epidemiological tool used to assess caries severity and prioritise treatment needs.^14^,^16^ Unlike conventional indices such as Decayed, Missed, Filled Teeth (DMFT) or International Caries Detection and Assessment System (ICDAS), CTNI categorises individuals according to treatment complexity, from preventive to surgical care, making it particularly useful for public health planning and resource allocation in population-based and resource-limited settings. In Argentina and other Latin American countries, CTNI has been used in school-based surveys to identify children at high risk of untreated decay and guide targeted interventions.^17^ Evidence shows that children from lower socio-economic backgrounds are more frequently classified in higher CTNI categories, reflecting a greater burden of untreated caries and limited access to care.

The present study was designed as a population study (preschool children living in Buenos Aires) for the purpose of assessing the CTNI and its ecological association with socio-economic indicators, as well as to evaluate a potential spatial correlation and plan health-related intervention.

## Materials and methods

This study employed an ecological design to explore the relationship between caries treatment needs and socio-economic and healthcare access variables among 6-year-old children attending public schools in the Autonomous City of Buenos Aires (CABA), Argentina.^16^ The protocol was approved by the Ethics Committee of the School of Dentistry, University of Buenos Aires (CETICA-FOUBA 023/2019) and complied with the legal regulations of the Ministry of Education of Buenos Aires City.

### Settings

The survey was carried out in public schools of Buenos Aires City (CABA) between 2017 and 2020.

**Figure 1:**
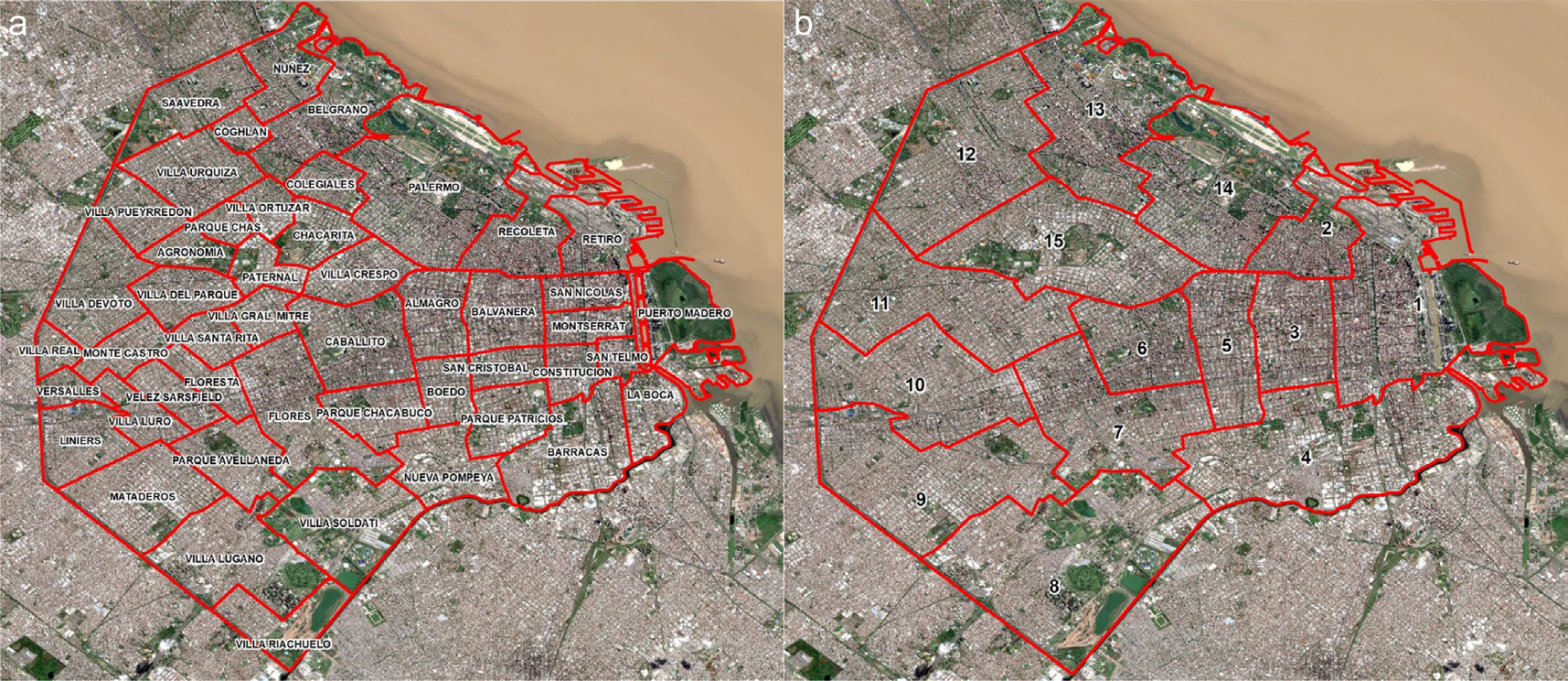
Administrative spatial units of Buenos Aires used in the analysis. (a) Official neighbourhoods (barrios); (b) Municipal divisions (comunas). The cartographic basemap was derived from Sentinel-2 imagery obtained from the Copernicus Data Space Ecosystem (Level-2A, 12 April 2026). Bands B04, B03 and B02 were combined to generate a natural-colour RGB composite (4-3-2), used as background for map visualisation. List of Municipalities and neighbourhoods (Barrios). Municipality 1, Barrios: Retiro, San Nicolás, Puerto Madero, San Telmo, Montserrat y Constitución. Municipality 2, Barrio: Recoleta. Municipality 3, Barrios: Balvanera y San Cristóbal. Municipality 4, Barrios: La Boca, Barracas, Parque Patricios y Nueva Pompeya. Municipality 5, Barrios: Almagro y Boedo. Municipality 6, Barrio: Caballito. Municipality 7, Barrios: Flores y Parque Chacabuco. Municipality 8, Barrios: Villa Soldati, Villa Riachuelo y Villa Lugano. Municipality 9, Barrios: Liniers, Mataderos y Parque Avellaneda. Municipality 10, Barrios: Villa Real, Monte Castro, Versalles, Floresta, Vélez Sarsfield y Villa Luro. Municipality 11, Barrios: Villa General Mitre, Villa Devoto, Villa del Parque y Villa Santa Rita. Municipality 12, Barrios: Coghlan, Saavedra, Villa Urquiza y Villa Pueyrredón. Municipality 13, Barrios: Núñez, Belgrano y Colegiales. Municipality 14, Barrio: Palermo. Municipality 15, Barrios: Chacarita, Villa Crespo, La Paternal, Villa Ortúzar, Agronomía y Parque Chas.

Buenos Aires is the capital of Argentina. The city proper has about 3.12 million inhabitants (as of the 2017 census) within its area of ∼205.9 km², while the Buenos Aires Metropolitan Area (Gran Buenos Aires) includes the city proper plus its surrounding urban belt and has around 15 million inhabitants. The city is administratively divided into 15 municipalities (*comunas*), and it is further subdivided into 48 official neighbourhoods (*barrios*) (figure 1a,b). The cartographic basemap was derived from satellite imagery obtained through the Copernicus Data Space Ecosystem, using a Sentinel-2B Level-2A scene acquired on 12 April 2026. Bands B04, B03 and B02, corresponding to the red, green and blue spectral channels, respectively, were extracted from the dataset; all bands had a spatial resolution of 10 m. The raster bands were imported into GIS software in JP2 format as separate layers and merged to produce a natural-colour RGB composite (4-3-2). The final image was visually optimised and used as the basemap for the maps presented in this study.

All the public primary schools within the city (n=471) were invited to participate, and 432 accepted (91.72%). The sampling frame was limited to public schools because the oral health surveillance programme and the CTNI-based activities were implemented through a formal cooperation agreement between the Ministry of Education and the Ministry of Health of Buenos Aires City, and the School of Dentistry of the University of Buenos Aires.

Children were included if they were enrolled in one of the participating public schools, had parental informed consent and provided verbal assent on the day of the clinical examination.

Children were excluded if their legal guardians did not provide authorisation; they attended schools located in areas where the safety of the fieldwork team could not be ensured; the school infrastructure did not meet minimum requirements to conduct the planned activities; they were receiving dental care through an ongoing oral health programme; or they were absent on the day of examination. Participation was voluntary.

### Patient and public involvement

Patients and members of the public were not involved in the design, conduct, analysis or reporting of this research. The study was based on data collected as part of a routine, city-wide oral health surveillance programme implemented in public schools in Buenos Aires by local health and education authorities. Parents or legal guardians provided written informed consent, and children gave verbal assent prior to participation. Although patients and the public did not actively contribute to the research process, the research question was informed by recognised public health priorities related to childhood oral health inequalities. The findings are intended to inform equitable public health planning and may support the development of targeted oral health interventions in socially disadvantaged communities.

### Clinical assessment and calibration procedures

A dental examination of children was conducted within the school environment in accordance with the criteria outlined in the CTNI.^15^ The examination was executed under controlled conditions, employing the utilisation of light, dental mirrors, WHO probes and magnification (2.5×). The dental examinations were carried out by 25 calibrated researchers. Before the commencement of the survey, the calibration process was conducted under the supervision of the reference examiner. The calibration strategy comprised three phases:

(First phase) A theoretical class (6 hours) with clinical photographs (n=48). The objective of this phase is to facilitate the recognition of the different categories in the CTNI.

(Second phase) The caries detection process was conducted on extracted teeth (n=210) using an ex vivo approach over 2 hours. The specimens were examined after drying the surfaces with compressed air and under adequate lighting. Each operator meticulously documented the observed findings according to the established lesion and activity criteria for each tooth surface. Subsequently, a discussion was held with the benchmark examiner regarding the results.

(Third phase) Clinical practice (30 hours). Each examiner was assigned a total of six volunteer children, who provided a balanced number of dental surfaces with CTNI codes. The findings were meticulously observed and documented in an ad hoc spreadsheet. The visual–tactile clinical examination was performed with a frontal light, WHO probes and air drying of the surfaces. After 1 week, a re-evaluation of each patient is conducted by the reference examiner, with the results of this evaluation documented. The reliability of the examination was assessed by comparing the results obtained from the benchmark and from each examiner. The percentage of agreement was determined using Cohen’s kappa statistic for healthy surfaces, enamel and dentinal lesions.^18^

### Socio-economic indicators

The socio-economic conditions were analysed using several proxies. The health coverage (PH_Cov) of each child was used as a socio-economical proxy at individual level; the housing price in US dollar (Hous_price) per square metre as a proxy at familiar level; the distance of the school to the nearest primary healthcare centre (CESAC) as proxy each at school level; the population density (Pob), derived from the National Census as proxy of socio-economical at neighbourhood (barrio) level; the percentage of households with unsatisfied basic needs (UBN) as proxy at neighbourhood level.^5^

### Data analysis

Data were organised and processed using Microsoft Excel (V.365; Microsoft Corporation, Redmond, Washington, USA), and statistical analyses were performed using Stata (V.18.5; StataCorp LLC, College Station, Texas, USA). The unit of analysis was considered the school/neighbourhood level and that inferences at the individual level were not considered. Exploratory data analysis was performed to characterise the distributions of CTNI range and socio-environmental variables and to examine preliminary associations among them. This step provides an initial assessment of key assumptions for subsequent linear modelling, such as normality and linearity of relationships.^19^ Histograms, Pearson correlations and scatterplots were used to detect patterns, outliers and distributional features that could affect model specification. One-way analysis of variance (ANOVA) and Pearson’s χ² were used appropriately. Multivariate regression models were built and run using the different socio-economic indicators (health coverage, housing price per square metres, the distance of the school to the nearest primary healthcare centre, population density and UBN) as dependent variables and the CTNI and neighbourhood of residence as covariates. Although the primary design of this study was ecological, using schools as the units of analysis, an individual level analysis was conducted to compare CTNI severity categories according to each child’s type of health coverage.

Spatial analysis required geographic localisation.^20^ Since individual residential addresses of children were not available, the school was selected as the unit of analysis. Public school georeferencing data were obtained from official spatial databases provided by the Government of CABA. All spatial data were reprojected into the Argentina Gauss-Krüger Buenos Aires coordinate reference system (EPSG:22185; Campo Inchauspe datum, Transverse Mercator projection) to ensure consistency in spatial calculations.

The subjects were stratified based on their CTNI as no needs (CTNI 0–2), low needs (CTNI 3–6), high needs (CTNI 7–10) and very high needs (CTNI 11–14).^15^ Consistent with the validation and comparative performance of CTNI reported in literature,^15^ thresholds of CTNI >2 and CTNI >10 were also used to identify, respectively, overall treatment needs and severe treatment needs. The association between CTNI categories and health coverage was tested via a matched case-control data procedure.

A spatial autoregressive (SAR) analysis quantified the extent to which variation in the dependent variables—CTNI and CTNI categories—was jointly determined by local socio-economic conditions and spatial dependence among neighbouring units. Geographical mapping and shapefile generation were performed using ArcGIS software (V.10.8.2; Esri, Redlands, California, USA) and QGIS software (V.3.38; QGIS.org, Grabs, Switz.).

The spatial structure of the data was first formalised through a contiguity- or distance-based spatial weights matrix, after which the dataset was declared as spatial. The spatial weights matrix (W) was generated using a row-standardised contiguity-based structure. Global Moran’s I was computed on residuals to assess spatial dependence. The multivariate SAR models incorporated a spatially lagged dependent variable to capture spillover effects, while socio-economic indicators served as covariates explaining local variation. This specification allowed estimation of both the direct effects of socio-economic factors on CTNI and the strength of spatial dependence, thereby assessing whether observed spatial patterns reflected substantive regional processes or underlying spatial autocorrelation. To enhance the credibility of the estimates, model fit and robustness were assessed using standard diagnostic procedures, including inspection of residual distributions, checks for multicollinearity and evaluation of residual spatial autocorrelation. Multicollinearity among socio-economic variables was assessed using variance inflation factors, and no critical multicollinearity issues were identified. The spatial lag specification corresponds to a SAR-lag model, and spatial relationships were defined using a row-standardised contiguity-based weights matrix.

## Results

A total of 471 public primary schools were scheduled for evaluation, and clinical examinations were completed in 432 (91.7%). In the remaining schools, assessments could not be performed due to operational constraints, including safety concerns and logistical limitations. These exclusions were geographically scattered and unlikely to introduce systematic bias. Among the children enrolled in the examined schools, those without consent, absent on the examination day or participating in dental care programmes were excluded, resulting in a final sample of 54 337 children. Inter-examiner reliability, measured by Cohen’s Kappa, was 0.75 (95% CI 0.72 to 0.81) while intraexaminer reliability was 0.83 (95% CI 0.79 to 0.88). Treatment needs prevalence (CTNI >2) was 67.94% of the sample, while high caries treatment needs (CTNI >10) were recorded in 9515 (17.51%) children; these figures were statistically dissimilar in the different neighbourhood (Pearson χ^2^_(14)_=2.5e+03 p<0.01 for caries treatment needs prevalence and Pearson χ^2^_(14)_ =1.7e+03 p<0.01 for high caries treatment needs, respectively) (online supplemental table 1s). The mean number of CTNI was 5.23±3.98 in Buenos Aires with a range from 3.69±3.29 in the municipality 13 (Belgrano, Coghlan and Nunez neighbourhoods) to municipality 8 (Villa Soldati, Villa Lugano and Villa Riacheulo neighbourhoods) (one-way ANOVA p<0.01) (table 1). All the socio-economic indicators were statistically dissimilar in the different neighbourhood (one-way ANOVA p<0.01 for housing price per square metres, population density, the distance of the school to the nearest primary healthcare centre and UBN and Pearson χ^2^ for health coverage). The descriptive analysis revealed substantial heterogeneity in caries treatment needs and socio-economic indicators across the neighbourhood of Buenos Aires (online supplemental table 2s). This spatial distribution mirrors pronounced disparities in health coverage: neighbourhoods with predominantly public health coverage exhibited higher CTNI values, whereas those with a larger share of private health insurance consistently showed lower treatment needs. Similarly, housing prices—a proxy for household socio-economic status—were markedly higher in northern municipalities (2, 3, 13, 14), reinforcing the gradient observed in oral health outcomes. Other structural indicators, such as population density and distance to the nearest primary health centre (CESAC), also demonstrated significant differences between municipalities (all p<0.01). The multivariate regression models (table 2) strengthened these observations by demonstrating significant inverse associations between CTNI and every socio-economic indicator examined, including private health coverage, housing price, CESAC proximity and population density (all p<0.01). Higher CTNI scores corresponded to lower socio-economic status, and the effect of commune of residence remained significant for most models, indicating that structural spatial inequalities persist even after adjusting for individual-level disease burden.

**Table 1:** Description of the population in terms of caries disease

	Caries treatment needs prevalence		Severe caries treatment needs	
	CTNI 0–2 n (%)	CTNI >2 n (%)	CTNI ≤11 n (%)	CTNI >11 n (%)
Buenos Aires	17 422 (32.06)	36 915 (67.94)	44 822 (82.49)	9515 (17.51)
Municipality 1	612 (29.69)	1449 (70.31)	1665 (80.79)	396 (19.21)
Municipality 2	336 (38.62)	534 (61.38)	761 (87.47)	109 (12.53)
Municipality 3	1152 (31.36)	2522 (68.64)	3097 (84.30)	577 (15.70)
Municipality 4	905 (22.70)	3082 (77.30)	3073 (77.08)	914 (22.92)
Municipality 5	1618 (62.62)	966 (37.38)	2205 (85.33)	379 (14,67)
Municipality 6	1280 (49.48)	1307 (50.52)	2359 (91.19)	228 (8.81)
Municipality 7	1132 (24.32)	3523 (75.68)	3601 (77.36)	1054 (22.64)
Municipality 8	1124 (16.24)	5799 (83.76)	4923 (71.11)	2000 (28.89)
Municipality 9	1127 (22.56)	3869 (77.44)	3799 (76.04)	1197 (23.96)
Municipality 10	1534 (32.89)	3130 (67.11)	3822 (81.95)	842 (18.05)
Municipality 11	1571 (40.35)	2322 (59.65)	3387 (87.00)	506 (13.00)
Municipality 12	1884 (38.46)	3.014 (61.54)	4369 (89.20)	529 (10.80)
Municipality 13	1237 (45.70)	1470 (54.30)	2512 (92.80)	195 (7.20)
Municipality 14	1186 (46.18)	1382 (53.82)	2321 (90.38)	247 (9.62)
Municipality 15	1376 (42.08)	1894 (57.92)	2928 (89.54)	342 (10.46)
	*Pearson χ^2^_(14)_=2.5e+03 p<0.01*		*Pearson χ^2^_(14)_=1.7e+03 p<0.01*	

List of municipalities and neighbourhoods (Barrios). Municipality 1, Barrios: Retiro, San Nicolás, Puerto Madero, San Telmo, Montserrat y Constitución. Municipality 2, Barrio: Recoleta. Municipality 3, Barrios: Balvanera y San Cristóbal. Municipality 4, Barrios: La Boca, Barracas, Parque Patricios y Nueva Pompeya. Municipality 5, Barrios: Almagro y Boedo. Municipality 6, Barrio: Caballito. Municipality 7, Barrios: Flores y Parque Chacabuco. Municipality 8, Barrios: Villa Soldati, Villa Riachuelo y Villa Lugano. Municipality 9, Barrios: Liniers, Mataderos y Parque Avellaneda. Municipality 10, Barrios: Villa Real, Monte Castro, Versalles, Floresta, Vélez Sarsfield y Villa Luro. Municipality 11, Barrios: Villa General Mitre, Villa Devoto, Villa del Parque y Villa Santa Rita. Municipality 12, Barrios: Coghlan, Saavedra, Villa Urquiza y Villa Pueyrredón. Municipality 13, Barrios: Núñez, Belgrano y Colegiales. Municipality 14, Barrio: Palermo. Municipality 15, Barrios: Chacarita, Villa Crespo, La Paternal, Villa Ortúzar, Agronomía y Parque Chas.

Caries treatment needs prevalence (CTNI score ≤2), severe caries treatment needs (CTNI >11) across living area (municipalities). The associations were assessed using the Pearson χ² test.

CTNI, Caries Treatment Needs Index.

**Table 2:** Multivariate regression models with socio-economic indicators as dependent variables: PH_Cov (proportion of public coverage), Hous_price (USD per m²), distance to CESAC (km), Pop (inhabitants per km²) and households with UBN (%)

	PH_Cov		Hous_price		CESAC		Pop		UBN	
	Coef (SE)	P value	Coef (SE)	P value	Coef (SE)	P value	Coef (SE)	P value	Coef (SE)	P value
CTNI	−0.04 (0.00)	<0.01	−5.57 (0.54)	<0.01	−19.71 (0.75)	<0.01	−362.32 (30.26)	<0.01	−0.01 (0.01)	<0.01
Municipality	0.02 (0.00)	<0.01	−0.32 (0.57)	0.58	33.99 (0.79)	<0.01	−1627.91 (31.96)	<0.01	−0.14 (0.01)	<0.01
Constant	1.52 (0.00)	<0.01	2550.98 (6.25)	<0.01	1109.48 (8.63)	<0.01	8184.22 (350.02)	<0.01	3.17 (0.02)	<0.01

Independent variables included CTNI and municipality of residence.

CESAC, primary healthcare centres; CTNI, Caries Treatment Needs Index; Hous_price, housing price; PH_Cov, health coverage; Pop, population density; UBN, unsatisfied basic needs.

The relationship between health coverage and caries treatment needs severity (categorised as no needs ‘CTNI 0–2’, low needs ‘CTNI 3–6’, high needs ‘CTNI 7–10’, very high needs ‘CTNI 11–14’) was further characterised through Mantel-Haenszel odds ratios (table 3). Children with public coverage exhibited progressively higher odds of belonging to worse CTNI categories, ranging from OR=1.77 for low needs (CTNI 3–6) to OR=3.68 for CTNI 11–14 very high needs (all p<0.01), with a strong dose–response trend (χ²=2668.05, p<0.01). This gradient underscores the cumulative disadvantage experienced by publicly insured populations.

**Table 3:** CTNI categories (no needs ‘CTNI 0–2’, low needs ‘CTNI 3–6’, high needs ‘CTNI 7–10’, very high needs ‘CTNI 11–14’) by health coverage (public or private)

CTNI	Health coverage		χ^2^	P value	OR	95% CI
	Public *n (%*)	Private *n (%*)				
No needs (*CTNI 0–2*)	6075 (34.87)	11 347 (65.13)			1.00	
Low needs (*CTNI 3–6*)	8815 (51.69)	8239 (48.31)	615.55	<0.01	1.77	1.69 to 1.85
High needs (*CTNI 7–10*)	6934 (67.02)	3412 (32.98)	1967.45	<0.01	3.03	2.87 to 3.21
Very high needs (*CTNI 11–14*)	6918 (72.71)	2597 (27.29)	2093.43	<0.01	3.68	3.46 to 3.90

Score for trend of odds: χ2_(1)_= 2668.05 p<0.01.

Mantel-Haenszel ORs adjusted for communes.

CTNI, Caries Treatment Needs Index.

**Figure 2:**
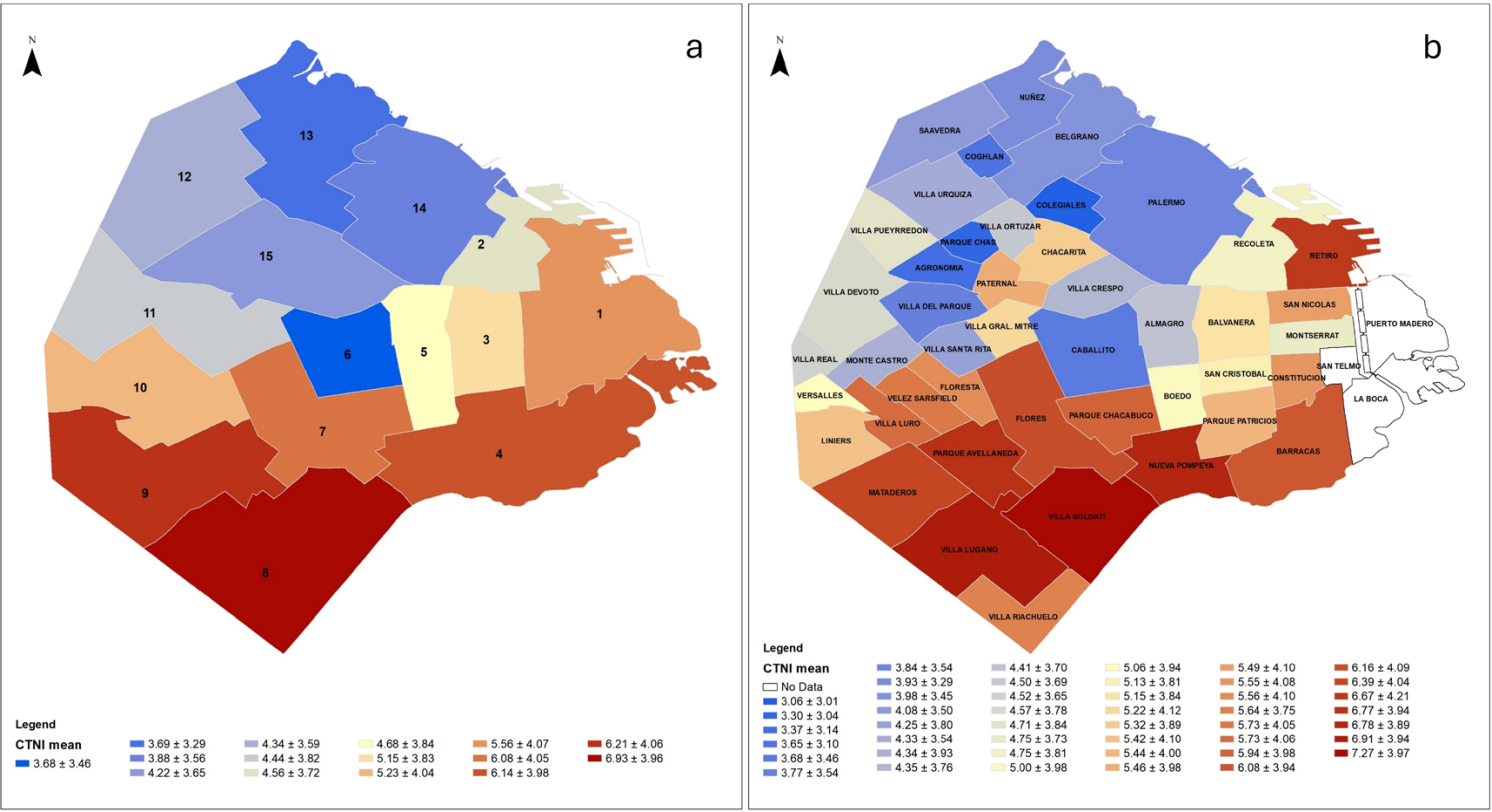
CTNI mean values and spatial distribution per municipality (*Comunas*, figure a) and neighbourhood (*Barrios*, figure b) of the CABA, Argentina. CABA, Autonomous City of Buenos Aires; CTNI, Caries Treatment Needs Index.

Finally, the SAR model (table 4) confirmed that socio-economic deprivation was significantly associated with CTNI distributions across municipalities (figure 2a,b). Lower health coverage rates, higher unmet basic needs and greater distance to health centres were all significant predictors of worse CTNI outcomes (p<0.01), while housing prices were non-significant.

**Table 4:** SAR-lag models by neighbourhood (Barrio)

a- CTNI as dependent variable and socio-economic indicators			
CTNI	Coef (SE)	P value	95% CI
Health Coverage	−2.60 (0.85)	<0.01	−4.27 to −0.94
UBN	−0.07 (0.01)	0.66	−0.38 to 0.24
Hous_price	0.03 (0.00)	<0.01	0.001 to 0.001
CESAC	−9.72e-06 (0.00)	0.97	−0.001 to −0.001
Constant	1.04 (0.56)		−0.06 to 2.15

b- Caries Treatment Needs categories as dependent variable and socio-economic indicators			
CTNI	Coef (SE)	P value	95% CI
Health Coverage	−0.49 (0.27)	0.06	−1.02 to 0.04
UBN	−0.19 (0.05)	<0.01	0.12 to 0.08
Hous_price	0.001 (0.00)	<0.01	0.001 to 0.001
CESAC	−2.35e-06 (0.00)	0.97	−0.001 to −0.001
Constant	0.33 (0.18)		−0.02 to 0.69

SAR model N observations = 48 neighbourhoods χ2_(4)_=67.90 p<0.01.

Moran test for spatial dependence. H0: error terms are i.i.d. Errorlags: W χ2(1)=20.82 p<0.01.

SAR model N observations = 48 neighbourhoods χ2(4)=111.08 p<0.01.

Moran test for spatial dependence. H0: error terms are i.i.d. Errorlags: W χ2(1)=20.02 p<0.01.

Independent variables included health coverage (proportion of public coverage), households with UBN (%), Hous_price (USD per m²) and distance to CESAC (km).

CESAC, primary health care centres; CTNI, Caries Treatment Needs Index; Hous_price, housing price; SAR, spatial autoregressive; UBN, unsatisfied basic needs.

## Discussion

This survey was designed to assess the geographical distribution of the caries treatment needs in children living in the capital of Argentina, Buenos Aires, and to evaluate which socio-economic indicator better fitted with the caries data. The outcomes offered across the descriptive, multivariate and spatial analyses collectively reveal a consistent and multidimensional pattern of socio-economic inequality underlying caries treatment needs among schoolchildren in Buenos Aires. These findings align with extensive literature showing that oral health indicators are not randomly distributed^21^,^28^ but instead mirror socio-territorial structures, reflecting how access, resources and living conditions shape disease burdens. The descriptive data clearly demonstrate a geographical clustering of higher CTNI scores within a neighbourhood characterised by lower socio-economic indicators. Municipalities with a predominance of public health coverage, lower housing prices, greater population density and higher prevalence of unmet basic needs consistently present higher levels of untreated caries. This suggests the existence of an ecological gradient where socio-economic disadvantage aggregates spatially and translates into poorer oral health outcomes.^29^,^31^

It is interesting to note the strength and consistency of the association between health coverage and caries severity. The Mantel-Haenszel analysis appears to indicate a noticeable dose–response relationship: as CTNI severity category increases, it seems that the proportion of children relying on public coverage grows substantially, and the odds of being in higher need categories escalate accordingly. This gradient is not merely a statistical phenomenon but reflects structural inequities in access to preventive and restorative dental care.^32 33^ In many contexts, private health coverage correlates with higher income, greater access to dental professionals and more regular use of preventive services. Conversely, households depending on public systems often encounter systemic barriers, including longer waiting times, fewer dental providers and reduced availability of specialised care. The observed disparities are therefore likely the result of cumulative social and systemic disadvantages rather than isolated individual behaviours.

The multivariate regression analyses deepen this interpretation by showing that CTNI is significantly associated with multiple socio-economic predictors even after adjusting for municipality of residence. This suggests that caries treatment needs are associated with both individual/family-level socio-economic factors (such as ability to afford private insurance or high-cost housing) and neighbourhood-level structural factors (such as population density or service accessibility). The negative associations of CTNI with housing price and population density are consistent with the notion that more affluent, less crowded neighbourhoods offer more favourable conditions for maintaining oral health, potentially through better availability of services, healthier food environments and reduced stressors associated with poverty.

The SAR model provides an even more nuanced understanding by demonstrating that spatial dependence—that is*,* the influence of neighbouring municipalities—plays an important role in shaping oral health patterns. The persistence of significant associations in this model indicates that communal socio-economic conditions exert contextual associations beyond individual circumstances. For example, living in areas with high unmet basic needs or limited access to primary healthcare facilities may restrict even motivated families from obtaining timely dental care. The spatial clustering implied by the SAR model also highlights that oral health inequalities are not randomly scattered but follow an identifiable territorial pattern, reflecting historical, economic and urban-planning dynamics.^33^,^35^

Taken together, these findings support the conceptualisation of dental caries not solely as a biological disease but as a socially distributed condition strongly shaped by structural determinants. They also illustrate the importance of adopting multilevel and spatial approaches in oral epidemiology to capture the complex interactions between individual, community and systemic factors. Importantly, these results provide evidence supporting targeted public health interventions. Strategies should prioritise high-needs municipalities, enhance accessibility of dental services in underserved areas and integrate oral health into broader social policies addressing poverty and inequality. In doing so, they could help reduce the substantial oral health inequities demonstrated across the city.

This spatial population study relied on area-level indicators; therefore, inferences at the individual level should be avoided (ecological fallacy). The results may also be affected by the modifiable areal unit problem (MAUP), as estimates can vary with the spatial scale and zoning of neighbourhoods/municipalities. Although the spatial models were estimated using an explicitly defined spatial-weights matrix (W) and standard diagnostic procedures (including residual Moran’s I), the results may still be affected by residual spatial heterogeneity and by the operational choice of W. As in all spatial epidemiological analyses, these specifications approximate rather than fully capture underlying spatial processes, and this should be considered when interpreting localised effects. Moreover, we did not have access to proximal, individual-level determinants of dental caries (dietary free sugars, oral-hygiene/fluoride practices, parental education and behaviours) recommended by international frameworks, nor to utilisation metrics (eg*,* visits, waiting times) that distinguish mere geographic availability from realised access—an important caveat in contexts where an inverse care law may operate. Finally, CTNI reflects treatment needs rather than the full natural history of disease; thus, effect estimates should be interpreted as area-level associations that inform territorial planning rather than individual risk. In fact, although CTNI is less commonly used internationally than DMFT or ICDAS, it provides a complementary and policy-relevant perspective by directly reflecting treatment complexity and service demand, which is particularly valuable for planning targeted public health interventions.

These findings should, however, be interpreted in light of some important methodological limitations. Its ecological design means the findings are vulnerable to ecological fallacy, so area-level associations should not be interpreted as individual-level risks. Several socio-economic variables were measured using proxies—such as health coverage, housing prices and school distance to primary care—which may only partly capture children’s true social and care contexts and may introduce misclassification. These indicators were selected to represent complementary dimensions of socio-economic context at different analytical levels. While proxies such as housing prices and population density may not directly reflect individual socio-economic status, they are commonly used contextual measures in spatial epidemiological research and help capture structural urban inequalities. Important confounders were unavailable, including dietary sugar intake, oral hygiene and fluoride exposure, parental education, oral health literacy and actual use of dental services. The absence of these behavioural and biological variables may have resulted in residual confounding, and therefore, the observed associations should be interpreted as contextual relationships rather than individual-level causal effects. In addition, using schools as the spatial unit rather than home addresses may have introduced spatial misclassification, and the results may be affected by the MAUP and residual spatial heterogeneity.

The findings of this study, although rooted in the specific social and urban landscape of Buenos Aires, have broad relevance for other metropolitan settings characterised by socio-economic stratification and uneven access to health services. Therefore, the observed gradients in CTNI can reasonably be generalised to comparable urban populations, highlighting the importance of integrating spatial and socio-economic perspectives into child oral health planning. Nonetheless, local contextual factors should be considered when applying these insights to different settings.

## Conclusion

This study demonstrates that caries treatment needs among preschool children in Buenos Aires are strongly patterned by socio-economic and territorial inequalities. Higher CTNI levels consistently clustered in municipalities marked by poverty, limited health coverage and reduced access to primary care services. Both individual disadvantage and neighbourhood context contributed to the burden of untreated caries, revealing oral health as a clear expression of broader social inequities. Targeted, area-based public health strategies—integrating oral health into primary care, strengthening services in underserved zones and addressing structural deprivation—are essential to reduce these disparities and improve children’s oral health outcomes across the city.

## Supplementary material

10.1136/bmjopen-2026-116230 online supplemental file 1

## Data Availability

Data are available upon reasonable request.
