# Supplementary material for: Spatial population study on the association among socio-economic indicators and oral health in preschool children in Buenos Aires
Source: BMJ Open. 2026 May 20;16(5):e116230. doi: 10.1136/bmjopen-2026-116230 (PMC13202111; doi:10.1136/bmjopen-2026-116230)
Supplement: online supplemental file 1 [file bmjopen-16-5-s001.docx]

**A Spatial Population Study on the association among Socio-Economic indicators and Oral Health in Preschool Children in Buenos Aires.**

Aldo Squassi, Eliana Belén González, Fiorella Ventura, Rocio Lazzati, Glenda Rossi, Pablo Salgado, Andrea Cappai, Marco Dettori, Guglielmo Campus

**Supplementary materials**

**Table 1.s** Description of the population in terms of caries disease. Caries treatment needs prevalence (CTNI score ≤2), Severe Caries Treatment Needs (CTNI >11) across living area (municipalities). The associations were assessed using the Pearson chi-square test.

**Figure 1.** Municipalities (*Comunas*, figure a) and neighborhoods (*Barrios*, figure b) bundaries of the Autonomous City of Buenos Aires (CABA), Argentina.

**Table 1s**. Description of the population in terms of caries disease. Caries treatment needs prevalence (CTNI score ≤2), Severe Caries Treatment Needs (CTNI >11) across living area (municipalities). The associations were assessed using the Pearson chi-square test.

|  | **Caries Treatment Needs Prevalence** | |  | **Severe Caries Treatment Needs** | |
| --- | --- | --- | --- | --- | --- |
|  | CTNI 0-2  n (%) | CTNI >2  n (%) |  | CTNI ≤11  n (%) | CTNI >11  n (%) |
| Buenos Aires | 17,422 (32.06) | 36,915 (67.94) |  | 44,822 (82.49) | 9,515 (17.51) |
|  |  |  |  |  |  |
| Municipality 1 | 612 (29.69) | 1,449 (70.31) |  | 1,665 (80.79) | 396 (19.21) |
| Municipality 2 | 336 (38.62) | 534 (61.38) |  | 761 (87.47) | 109 (12.53) |
| Municipality 3 | 1,152 (31.36) | 2,522 (68.64) |  | 3,097 (84.30) | 577 (15.70) |
| Municipality 4 | 905 (22.70) | 3,082 (77.30) |  | 3,073 (77.08) | 914 (22.92) |
| Municipality 5 | 1,618 (62.62) | 966 (37.38) |  | 2,205 (85.33) | 379 (14,67) |
| Municipality 6 | 1,280 (49.48) | 1,307 (50.52) |  | 2,359 (91.19) | 228 (8.81) |
| Municipality 7 | 1,132 (24.32) | 3,523 (75.68) |  | 3,601 (77.36) | 1,054 (22.64) |
| Municipality 8 | 1,124 (16.24) | 5,799 (83.76) |  | 4,923 (71.11) | 2,000 (28.89) |
| Municipality 9 | 1,127 (22.56) | 3,869 (77.44) |  | 3,799 (76.04) | 1,197 (23.96) |
| Municipality 10 | 1,534 (32.89) | 3,130 (67.11) |  | 3,822 (81.95) | 842 (18.05) |
| Municipality 11 | 1,571 (40.35) | 2,322 (59.65) |  | 3,387 (87.00) | 506 (13.00) |
| Municipality 12 | 1,884 (38.46) | 3.014 (61.54) |  | 4,369 (89.20) | 529 (10.80) |
| Municipality 13 | 1,237 (45.70) | 1,470 (54.30) |  | 2,512 (92.80) | 195 (7.20) |
| Municipality 14 | 1,186 (46.18) | 1,382 (53.82) |  | 2,321 (90.38) | 247 (9.62) |
| Municipality 15 | 1,376 (42.08) | 1,894 (57.92) |  | 2,928 (89.54) | 342 (10.46) |
|  | *Pearson χ^2^_(14)_=2.5e+03 p<0.01* | |  | *Pearson χ^2^_(14)_=1.7e+03 p<0.01* | |

*List of Municipalities and neighborhoods (Barrios). Municipality 1, Barrios: Retiro, San Nicolás, Puerto Madero, San Telmo, Montserrat y Constitución. Municipality 2, Barrio: Recoleta. Municipality 3, Barrios: Balvanera y San Cristóbal. Municipality 4, Barrios: La Boca, Barracas, Parque Patricios y Nueva Pompeya. Municipality 5, Barrios: Almagro y Boedo. Municipality 6, Barrio: Caballito. Municipality 7, Barrios: Flores y Parque Chacabuco. Municipality 8, Barrios: Villa Soldati, Villa Riachuelo y Villa Lugano. Municipality 9, Barrios: Liniers, Mataderos y Parque Avellaneda. Municipality 10, Barrios: Villa Real, Monte Castro, Versalles, Floresta, Vélez Sarsfield y Villa Luro. Municipality 11, Barrios: Villa General Mitre, Villa Devoto, Villa del Parque y Villa Santa Rita. Municipality 12, Barrios: Coghlan, Saavedra, Villa Urquiza y Villa Pueyrredón. Municipality 13, Barrios: Núñez, Belgrano y Colegiales. Municipality 14, Barrio: Palermo. Municipality 15, Barrios: Chacarita, Villa Crespo, La Paternal, Villa Ortúzar, Agronomía y Parque Chas.*

**Table 2s**. Descriptive statistics of treatment needs of caries disease (CTNI) and socioeconomic factors: Health Coverage (PH_Cov, % public/private) at individual level; housing price (Hous_price, USD per m²) at household level; distance to the nearest Primary Health Care Center (CESAC, km) at school level; population density (Pop, inhabitants per km²) and households with Unsatisfied Basic Needs (UBN, %) at neighbourhood level. Differences between municipalities were assessed using one-way ANOVA or Pearson’s chi-square test.

|  | CTNI | | PH_COV | | Hous_price | | CESAC | Pop | UBN |
| --- | --- | --- | --- | --- | --- | --- | --- | --- | --- |
|  | *Mean±SD* | Private  *n (%)* | | Public  *n (%)* | | *Mean±SD* | *Mean±SD* | *Mean±SD* | *Mean±SD* |
| Buenos Aires | 5.23±3.98 | 25,595 (47.10) | | 28,742 (52.90) | | 2,519.09± 498.28 | 1.29±0.70 | 66,151.38±28,554.52 | 1.88±1.58 |
|  |  |  | |  | |  |  |  |  |
| Municipality 1 | 5.56±4.07 | 731 (35.47) | | 1,330 (64.53) | | 2,839.53±443.20 | 1.50±0.42 | 57,154.14±16,909.44 | 3.65±4.21 |
| Municipality 2 | 4.56±3.72 | 467 (53.68) | | 403 (46.32) | | 2,587.24±377.35 | 1.22±0.50 | 83,201.78±38,879.16 | 4.47±3.77 |
| Municipality 3 | 5.15±3.83 | 1,659 (45.16) | | 2,015 (54.84) | | 2,417.76±371.82 | 1.23±0.51 | 90,497.86±38,790.08 | 3.40±2.20 |
| Municipality 4 | 6.14±3.98 | 1,365 (34.24) | | 2,622 (65.76) | | 2,601.49±467.25 | 0.93±0.42 | 85,749.04±31,091.03 | 2.83±2.03 |
| Municipality 5 | 4.68±3.84 | 1,517 (58.71) | | 1,067 (41.29) | | 2,562.25±533.85 | 0.96±0.31 | 81,990.97±40,843.19 | 2.04±1.08 |
| Municipality 6 | 3.68±3.41 | 1,919 (74.18) | | 668 (25.82) | | 2,732.80±485.06 | 1.86±0.40 | 82,549.72±27,291.69 | 1.60±0.74 |
| Municipality 7 | 6.08±4.05 | 1,573 (33.79) | | 3,082 (66.21) | | 2,467.10±627.69 | 1.36±0.67 | 67,642.26±23,383.62 | 1.44±0.60 |
| Municipality 8 | 6.93±3.96 | 1,471 (21.25) | | 5,452 (78.75) | | 2,311.05±599.38 | 0.54±0.33 | 61,147.05±21,934.94 | 1.34±0.75 |
| Municipality 9 | 6.21±4.06 | 1,569 (31.41) | | 3,427 (68.59) | | 2,398.11±341.22 | 1.29±0.67 | 45,285.32±14,677.59 | 1.44±0.55 |
| Municipality 10 | 5.23±4.03 | 2,313 (49.59) | | 2,351 (50.41) | | 2,458.75±264.33 | 1.63±0.91 | 54.693.90±13.358.69 | 1.45±0.53 |
| Municipality 11 | 4.44±3.82 | 2,339 (60.08) | | 1,554 (39.92) | | 2,519.99±384.34 | 1.67±0.90 | 52.548.65±15.361.92 | 1.40±0.69 |
| Municipality 12 | 4.33±3.59 | 3,098 (63.25) | | 1,800 (36.75) | | 2,542.92±456.68 | 1.12±0.39 | 51,496.61±17,523.38 | 1.58±0.55 |
| Municipality 13 | 3.69±3.29 | 1,793 (66.24) | | 914 (33.76) | | 2668.63±554.11 | 1.89±0.67 | 69,891.79±20,320.78 | 1.51±0.57 |
| Municipality 14 | 3.88±3.56 | 1,727 (67.25) | | 841 (32.75) | | 2660.09±565.17 | 1.59±0.64 | 88,796.83±32,791.28 | 2.09±0.92 |
| Municipality 15 | 4.22±3.65 | 2,054 (62.81) | | 1,216 (37.19) | | 2623.40±497.64 | 1.63±0.40 | 64,761.66±18,574.79 | 1.56±0.69 |
|  | *p<0.01*^*^ | *p<0.01*^§^ | | | *p<0.01*^*^ | | *p<0.01*^*^ | *p<0.01*^*^ | *p<0.01*^*^ |

^*^Analysis of Variance One -way; ^§^Pearson chi-square

*List of Municipalities and neighborhoods (Barrios). Municipality 1, Barrios: Retiro, San Nicolás, Puerto Madero, San Telmo, Montserrat y Constitución. Municipality 2, Barrio: Recoleta. Municipality 3, Barrios: Balvanera y San Cristóbal. Municipality 4, Barrios: La Boca, Barracas, Parque Patricios y Nueva Pompeya. Municipality 5, Barrios: Almagro y Boedo. Municipality 6, Barrio: Caballito. Municipality 7, Barrios: Flores y Parque Chacabuco. Municipality 8, Barrios: Villa Soldati, Villa Riachuelo y Villa Lugano. Municipality 9, Barrios: Liniers, Mataderos y Parque Avellaneda. Municipality 10, Barrios: Villa Real, Monte Castro, Versalles, Floresta, Vélez Sarsfield y Villa Luro. Municipality 11, Barrios: Villa General Mitre, Villa Devoto, Villa del Parque y Villa Santa Rita. Municipality 12, Barrios: Coghlan, Saavedra, Villa Urquiza y Villa Pueyrredón. Municipality 13, Barrios: Núñez, Belgrano y Colegiales. Municipality 14, Barrio: Palermo. Municipality 15, Barrios: Chacarita, Villa Crespo, La Paternal, Villa Ortúzar, Agronomía y Parque Chas.*
