# Supplementary material for: Spatial population study on the association among socio-economic indicators and oral health in preschool children in Buenos Aires
Source: BMJ Open. 2026 May 20;16(5):e116230. doi: 10.1136/bmjopen-2026-116230 (PMC13202111; doi:10.1136/bmjopen-2026-116230)
Supplement: Supplementary file 2 [file bmjopen-2026-116230.reviewer_comments.pdf]

PEER REVIEW HISTORY

BMJ Open publishes all reviews undertaken for accepted manuscripts. Reviewers are asked to complete a checklist review form and are provided with free text boxes to elaborate on their assessment. These free text comments are reproduced below.

ARTICLE DETAILS

Title (Provisional)

A Spatial Population Study on the association among Socio-Economic indicators and Oral Health in Preschool Children in Buenos Aires

Authors

Squassi, Aldo; Belén González, Eliana; Ventura, Fiorella; Lazzati, Rocio; Rossi, Glenda; Salgado, Pablo; Cappai, Andrea; Dettori, Marco; Campus, Guglielmo

---

VERSION 1 - REVIEW

**Reviewer**	**1**
**Name**	**Ribas-Pérez , David**
**Affiliation**	**Universidad de Sevilla, Stomatology**
**Date**	**26-Feb-2026**
**COI**	**None**

This manuscript addresses a highly relevant public health issue: the socioeconomic and spatial distribution of dental caries treatment needs among preschool children in Buenos Aires. The very large sample size (n = 54,337) and the integration of spatial autoregressive modelling represent important methodological strengths. The topic is timely and policy-relevant. However, several conceptual and reporting aspects could be strengthened to enhance clarity, precision, and scientific robustness.

The ecological design should be clearly emphasized as such (risk of ecological fallacy).

Specify whether CTNI thresholds (e.g., >2, >10) are validated cut-offs.

Odds ratio ranges (1.77–3.68) need clearer explanation: do these represent different CTNI categories?

Brief mention of model fit or robustness would enhance credibility.

The phrase "independently increasing needs" should be rephrased as "independently associated with increased needs."

This is a strong population-based public health study with clear epidemiological and policy relevance. The integration of spatial analysis and treatment complexity indices represents a valuable contribution.

**Reviewer**	**2**
**Name**	**Detsomboonrat, Palinee**
**Affiliation**	**Chulalongkorn University**
**Date**	**16-Mar-2026**
**COI**	**None**

This manuscript presents a large population-based ecological study examining the association between socioeconomic indicators and caries treatment needs among preschool children in Buenos Aires. The study includes a very large sample (n=54,337) and applies spatial analysis techniques to explore geographic inequalities in oral health. The topic is relevant for public health and oral epidemiology, and the integration of spatial analysis with treatment-need indices is potentially valuable.

However, several conceptual, methodological, and reporting issues require clarification before the manuscript can be considered for publication. In particular, the ecological design, definition of socioeconomic proxies, statistical modelling strategy, and interpretation of findings require further justification and clearer reporting. Some sections also contain inconsistencies in terminology, grammar, and variable definitions.

Major Comments

1. Study design and unit of analysis

The manuscript describes the study as an ecological population study, yet several analyses appear to be conducted at the individual level (e.g., odds ratios comparing health coverage categories). This creates conceptual inconsistency.

The authors should clearly specify:

What is the primary unit of analysis (individual child, school, or municipality)?

Which analyses were performed at individual level vs. area level.

How ecological and individual-level analyses were integrated.

Without clarification, there is a risk of methodological ambiguity and potential ecological fallacy.

2. Selection bias and representativeness

The sample includes only children attending public schools, while children attending private schools were not included. Since socioeconomic status is strongly associated with school type, this exclusion may bias estimates of socioeconomic gradients.

3. Use of CTNI as the primary outcome

The manuscript relies on the Caries Treatment Needs Index (CTNI) as the main measure of oral health. While CTNI may be useful for treatment planning, it is less commonly used internationally compared with dmft/DMFT or ICDAS-based indicators.

4. Definition and validity of socioeconomic indicators

Several socioeconomic proxies are used, including: health coverage, housing price per square meter, distance to primary health centres, population density. However, the rationale for selecting these indicators and their conceptual level (individual, household, neighbourhood) is not sufficiently explained.

Examaple: Housing price as a proxy for family socioeconomic status may not accurately reflect household income. Population density may reflect urban structure rather than socioeconomic deprivation.

The authors should provide stronger conceptual justification and references supporting these proxies.

5. Spatial analysis methodology

The manuscript reports the use of spatial autoregressive (SAR) models, but key methodological details are insufficiently described.

The authors should clarify:

Whether multicollinearity among socioeconomic variables was assessed.

More transparent reporting is necessary to allow replication and proper interpretation.

6. Interpretation of associations

Some parts of the discussion appear to imply causal interpretations, which are not supported by the cross-sectional ecological design.

For example, statements suggesting that socioeconomic deprivation "leads to" or "results in" worse CTNI outcomes should be interpreted cautiously.

The authors should revise the discussion to emphasize:

Associations rather than causal relationships

The limitations of cross-sectional and ecological analyses

7. Lack of key behavioural and biological variables

Important determinants of dental caries were not included in the analysis, including: sugar consumption, oral hygiene practices, fluoride exposure, parental education

While the authors acknowledge this limitation briefly, the potential impact on residual confounding should be discussed more thoroughly.

Minor Comments

1. Language and grammar

Several sentences require grammatical revision. Examples include:

"The examination was executed under controlled conditions" → consider simplifying to "The examinations were conducted under standardized conditions."

A thorough language editing is recommended.

2. Terminology consistency

There are inconsistencies in variable names throughout the manuscript:

Examples:

CTNI vs CNIT

CYNI appears in one section (likely a typographical error)

These should be corrected for clarity.

3. Clarification of inclusion/exclusion criteria

The manuscript states that children were excluded if:

"they attended schools located in areas where the safety of the fieldwork team could not be ensured."

The authors should briefly explain:

How many schools were excluded for this reason.

Whether this may introduce systematic bias.

4. Calibration procedures

The calibration process is described, but the manuscript does not report the kappa values obtained. Reporting the actual reliability statistics would strengthen the methodological transparency.

5. Tables and figures

Some tables are difficult to interpret due to formatting and unclear variable definitions. For example:

Table 2 includes several abbreviations that are not fully explained.

Units for housing prices and population density should be clearly stated.

VERSION 1 - AUTHOR RESPONSE

Reviewer 1

This manuscript addresses a highly relevant public health issue: the socioeconomic and spatial distribution of dental caries treatment needs among preschool children in Buenos Aires. The very large sample size (n = 54,337) and the integration of spatial autoregressive modelling represent

important methodological strengths. The topic is timely and policy-relevant. However, several conceptual and reporting aspects could be strengthened to enhance clarity, precision, and scientific robustness.
We sincerely thank the Reviewer for the positive and constructive evaluation. We appreciate the recognition of the large population-based dataset and the integration of spatial analysis, as well as the valuable suggestions aimed at improving clarity and methodological transparency. We have carefully addressed all the points raised and revised the manuscript accordingly, as detailed below.

The ecological design should be clearly emphasized as such (risk of ecological fallacy).
We thank the Reviewer for highlighting this important point. The ecological nature of the study and the associated risk of ecological fallacy have now been more explicitly emphasized both in the Methods and in the Discussion sections. We have clarified that the unit of analysis is the school/neighbourhood level and that inferences at the individual level should be avoided. Additionally, we expanded the discussion of ecological limitations to improve transparency and interpretability of the findings.

2. CTNI cut-offs
Specify whether CTNI thresholds (e.g., >2, >10) are validated cut-offs.
We appreciate this observation. The CTNI thresholds used in the study (e.g., CTNI 0–2, 3–6, 7–10, and 11–14) are based on previously published and validated categorizations of treatment needs, as reported in the original CTNI methodological literature. We have now clarified this in the Methods section and added an explicit reference to support the validity of these cut-offs.

3. Odds ratios clarification
Odds ratio ranges (1.77–3.68) need clearer explanation: do these represent different CTNI categories?
The reported odds ratios refer to the association between type of health coverage and increasing CTNI severity categories (low, high, and very high treatment needs), derived from Mantel–Haenszel analysis. We have now clarified this in the Results section to explicitly indicate that these values represent progressively higher odds of belonging to more severe CTNI categories.

4. Model fit and robustness
Brief mention of model fit or robustness would enhance credibility.
We appreciate this suggestion. Although the primary focus of the analysis was on association patterns, we have now included a brief description of model diagnostics and robustness checks in the Methods section. Specifically, we wrote "To enhance the credibility of the estimates, model fit and robustness were assessed using standard diagnostic procedures, including inspection of residual distributions, checks for multicollinearity, and evaluation of residual spatial autocorrelation."

5. Causal language
The phrase "independently increasing needs" should be rephrased as "independently associated with increased needs."
We fully agree. The phrasing has been revised throughout the manuscript to avoid causal interpretations and to ensure consistency with the observational ecological design. Specifically, expressions such as "independently increasing needs" have been replaced with "independently associated with increased needs."

This is a strong population-based public health study with clear epidemiological and policy relevance. The integration of spatial analysis and treatment complexity indices represents a valuable contribution.

We thank the Reviewer for the positive overall assessment of the study and for recognizing its epidemiological and policy relevance. We believe that the revisions have strengthened the clarity and methodological transparency of the manuscript.

Reviewer 2

This manuscript presents a large population-based ecological study examining the association between socioeconomic indicators and caries treatment needs among preschool children in Buenos Aires. The study includes a very large sample (n=54,337) and applies spatial analysis techniques to explore geographic inequalities in oral health. The topic is relevant for public health and oral epidemiology, and the integration of spatial analysis with treatment-need indices is potentially valuable.
However, several conceptual, methodological, and reporting issues require clarification before the manuscript can be considered for publication. In particular, the ecological design, definition of socioeconomic proxies, statistical modelling strategy, and interpretation of findings require further justification and clearer reporting. Some sections also contain inconsistencies in terminology, grammar, and variable definitions.
We sincerely thank the Reviewer for the detailed and insightful evaluation of our manuscript. We appreciate the recognition of the relevance of the topic and the value of integrating spatial analysis with treatment-need indices. We have carefully addressed all the comments raised, with particular attention to improving conceptual clarity, methodological transparency, and consistency in reporting. The manuscript has been substantially revised accordingly, as detailed below.

1. **Study design and unit of analysis**

The manuscript describes the study as an ecological population study, yet several analyses appear to be conducted at the individual level (e.g., odds ratios comparing health coverage categories). This creates conceptual inconsistency.
Thanks, for this important observation. The primary design of the study is ecological, with schools (and their corresponding neighbourhood context) as the unit of analysis. However, individual-level variables (e.g., type of health coverage) were available and were used in specific analyses to complement the ecological framework.
We clarified in the Methods section that: - The primary analytical framework is ecological (school/neighbourhood level); - Individual-level analyses (e.g., Mantel–Haenszel odds ratios) were conducted as complementary analyses to explore gradients within the population; - No causal inference at the individual level is intended.
This clarification aims to resolve the apparent inconsistency and improve methodological transparency.

2. Selection bias and representativeness

The sample includes only children attending public schools, while children attending private schools were not included. Since socioeconomic status is strongly associated with school type, this exclusion may bias estimates of socioeconomic gradients.
The inclusion of only public schools reflects the structure of the oral health surveillance program implemented by the Ministry of Health and Education of Buenos Aires, which operates within the public school system. We agree that this may limit representativeness, particularly with respect to higher socioeconomic groups attending private schools.
We have now explicitly acknowledged this as a potential source of selection bias in the Discussion section and clarified that the findings are most representative of children within the public education system, who also represent the most vulnerable segment of the population from a public health perspective.

3. Use of CTNI

The manuscript relies on the Caries Treatment Needs Index (CTNI) as the main measure of oral health. While CTNI may be useful for treatment planning, it is less commonly used internationally compared with dmft/DMFT or ICDAS-based indicators.

We appreciate this observation. The CTNI was selected because it provides a treatment-oriented classification of caries severity, which is particularly relevant for public health planning and resource allocation. While indices such as DMFT and ICDAS are widely used for epidemiological comparisons, CTNI offers a complementary perspective focused on treatment complexity and service needs.

In the revised Introduction, we clarified that CTNI complements conventional indices such as DMFT and ICDAS by classifying treatment complexity and service needs. In the Discussion, we further emphasized that CTNI offers a policy-relevant perspective for planning targeted oral health interventions and resource allocation.

4. Socioeconomic indicators

Several socioeconomic proxies are used… However, the rationale for selecting these indicators and their conceptual level… is not sufficiently explained.

We have now expanded the Methods section to provide a clearer conceptual justification for each socioeconomic indicator, specifying their analytical level (individual, household, or neighbourhood) and their relevance as proxies of socioeconomic conditions.

We also acknowledge that some proxies (e.g., housing price, population density) may imperfectly capture socioeconomic status and may reflect broader urban structural characteristics. This limitation has been explicitly discussed in the revised manuscript.

5. Spatial analysis and multicollinearity

The manuscript reports the use of spatial autoregressive (SAR) models, but key methodological details are insufficiently described… Whether multicollinearity among socioeconomic variables was assessed.

We appreciate this important methodological observation. We have expanded the Methods section to provide additional details on the spatial modelling approach, including clarification of the SAR specification and the construction of the spatial weights matrix.

Furthermore, we have now clarified that multicollinearity among socioeconomic variables was assessed using standard diagnostic procedures (e.g., variance inflation factors), and no critical multicollinearity issues were identified. These additions aim to improve transparency and reproducibility of the analytical approach.

6. Interpretation of associations

Some parts of the discussion appear to imply causal interpretations…

We fully agree with the Reviewer. The manuscript has been revised throughout to avoid causal interpretations and to ensure consistency with the cross-sectional ecological design. All statements suggesting causality have been reformulated to reflect associations.

7. Missing variables

Reviewer comment: "Important determinants… were not included…"

Response: We thank the Reviewer for this comment. We have expanded the Discussion section to more thoroughly address the absence of key behavioural and biological variables (e.g., diet, oral hygiene, fluoride exposure, parental education), and to acknowledge the potential for residual confounding. We also clarified that the study focuses on structural and contextual determinants rather than proximal individual behaviours.

Minor Comments

1. Language

Reviewer comment: “Several sentences require grammatical revision…”

Response: We thank the Reviewer for this suggestion. The manuscript has undergone thorough language editing to improve clarity, readability, and grammatical consistency.

2. Terminology

Inconsistencies in variable names…

Response: We thank the Reviewer for noting these inconsistencies. All variable names and abbreviations have been carefully reviewed and corrected throughout the manuscript.

3. Inclusion/exclusion criteria

Reviewer comment: “The manuscript states that children were excluded…”

Response: We thank the Reviewer for this comment. We have clarified in the Methods section the number of schools excluded due to safety concerns and discussed the potential implications for representativeness.

4. Calibration

Reviewer comment: “The manuscript does not report the kappa values…”

Response: We appreciate this observation. We have now included the kappa values obtained during calibration procedures in the Methods section to improve methodological transparency.

5. Tables

Reviewer comment: “Some tables are difficult to interpret…”

Response: We thank the reviewer for this helpful comment. Tables have been revised to improve clarity. Abbreviations have been standardized throughout, and units for socioeconomic indicators (housing price, population density, distance to primary health care centres, and Unsatisfied Basic Needs) have been explicitly specified in the table captions. Variable definitions have also been clarified to facilitate interpretation.
